# In vitro activity of *Morinda citrifolia* Linn. fruit juice against the axenic amastigote form of *Leishmania amazonensis* and its hydrogen peroxide induction capacity in BALB/c peritoneal macrophages

**DOI:** 10.1186/s13104-018-3555-7

**Published:** 2018-07-18

**Authors:** Fernando Almeida-Souza, Ana Elisa Reis de Oliveira, Ana Lucia Abreu-Silva, Kátia da Silva Calabrese

**Affiliations:** 1 Universidade Estadual do Maranhão, São Luís, Maranhão Brazil; 2 Laboratório de Imunomodulação e Protozoologia, Instituto Oswaldo Cruz, Fiocruz, Rio de Janeiro, 21040-900 Brazil

**Keywords:** Leishmaniasis, MTT, Phytotherapy, Antileishmanial activity, H_2_O_2_, Reactive oxygen species, Noni

## Abstract

**Objective:**

The current treatment of leishmaniasis induces strong side effects and increasing numbers of cases of resistance to reference drugs have been reported. The discovery of the therapeutic properties of active substances in plant extracts represents an interesting field of research into a more efficient treatment against leishmaniasis. *Morinda citrifolia*, commonly known as noni, has demonstrated promising results as antileishmanial and immunomodulator. Thus, the aim of this work was to evaluate activity against axenic amastigote and hydrogen peroxide induction capacity by *M. citrifolia* fruit juice.

**Results:**

Phytochemical screening identified anthraquinones, flavonoids, alkaloids, terpenoids, steroids, saponins, coumarins, phenolic compounds, tannins, anthocyanidins and chalcones. Noni juice exhibited dose-dependent activity and an IC_50_ of 240.1 µg/mL for axenic amastigotes. An absence of endotoxins was observed at the concentrations analyzed, while no cytotoxic effects were identified. Noni juice induced hydrogen peroxide production in BALB/c peritoneal macrophages but not in macrophages infected with *Leishmania amazonensis*. *M. citrifolia* fruit juice exhibited antileishmanial activity against *L. amazonensis* axenic amastigotes and activated macrophages by hydrogen peroxide induction, asserting its potential for further research into new forms of leishmaniasis treatment.

## Introduction

Leishmaniasis treatment currently uses pentavalent antimonials, amphotericin B or pentamidine, which are expensive and involve parenteral administration for prolonged periods, causing discomfort to patients. Several side effects have been reported, such as arthralgia, myalgia, cardiotoxicity, hepatotoxicity, nephrotoxicity, anorexia, nausea and vomiting [1, 2]. These side effects and the increasing number of cases of resistance to current treatments against leishmaniasis demonstrate the need for research into new treatment options which are more effective and less toxic. Such studies have been performed around the world to identify and characterize herbal extracts with antileishmanial activity [3, 4], such as *Morinda citrifolia* fruit juice.

*Morinda citrifolia*, known as ‘noni’, is native of Indian Ocean and Polynesia [5] and widely distributed around the world. The plant quickly adapted to different biomes in Brazil, including the Amazon rainforest, and their pharmacological properties as antimicrobial, antifungal and antitumor has been well described [6]. Our previous study with *M. citrifolia* fruit juice found in vitro antileishmanial activity against *L. infantum chagasi* [7] and *Leishmania amazonensis* [8], however, the activity against axenic amastigote has not yet been elucidated. The activity against axenic amastigote form allows evaluate the direct action of drugs without interference of mechanisms dependent of macrophage activation and disregarding its permeability in cell membranes. Activity against *L. amazonensis* correlated macrophage activation and nitric oxide (NO) induction by *M. citrifolia* [8], although the association between iNOS expression and the decrease of the parasite load was not observed in noni-treated mice, suggesting a different mechanism for killing parasites in vivo [9]. Moreover, the role of other mechanisms of macrophage activation by *M. citrifolia* fruit juice remains unknown.

The induction of reactive oxygen and nitrogen species is an important mechanism used by mammalian hosts against intracellular microbial pathogens, as *Leishmania*. Hydrogen peroxide (H_2_O_2_) is a major source of hydroxyl radicals and other reactive oxygen species (ROS), which macrophages produce in greater quantities [10, 11]. The present study therefore aimed to evaluate the phytochemical composition of *M. citrifolia* fruit juice, its in vitro antileishmanial activity against the axenic amastigote form of *L. amazonensis,* and its ability to induce H_2_O_2_ in peritoneal macrophages from BALB/c mice.

## Main text

### Materials and methods

#### Plant material

*Morinda citrifolia* fruit juice were obtained from plants of São Luís, Maranhão, Amazon region has described before [7]. The plant material was properly identified by Ana Maria Maciel Leite, and the voucher specimen, number 2000346, was deposited at the Herbarium Professora Rosa Mochel of the State University of Maranhão. The collected fruits were washed with distilled and sterilized water, dried and placed in sterile glass bottles for 3 days to drain off the juice released. The juice was centrifuged twice at 4000 rpm for 15 min; then the supernatant was lyophilized and stored at − 20 °C. The juice was diluted in culture medium and filtered through a 0.22 µm membrane for biological testing.

#### Phytochemical analysis

A quantity of 100 mg of *M. citrifolia* fruit juice was solubilized in 5 mL of methanol and used to identify class of chemical constituents in accordance with techniques adapted from Costa [12] and Matos [13]. After reaction with solutions described in Table 1, anthraquinones, anthocyanidins and chalcones were identified by red staining; flavonoids and coumarins were identified by fluorescence; alkaloids were identified by white turbidity; triterpenoids and steroids were identified by the greenish and red; saponins were identified by the presence of foam after shaking; phenolic compounds were identified by dark blue stain on filter paper; tannins were identified by white precipitate.

**Table 1:** Phytochemical screening of *Morinda citrifolia* fruit juice

Chemical constituents	Reactions	Results
Anthraquinone	NaOH	+
Flavonoids	AlCl_3_	+
Alkaloids	Mayer	+
	Bouchardat	+
	Hager	+
Triterpenes and steroids	Anhydride acetic and sulfuric acid	+
Saponins	Foam index	+
Coumarins	KOH	+
Phenolic compounds	FeCl_3_	+
Tannins	Gelatin	+
Anthocyanins	HCl	+
	NaOH	+
Chalcones	HCl	+
	NaOH	−

According to Costa (1992) [12] and Matos [13]

(+) presence; (−) absence

#### Parasite

The promastigote forms of *L. amazonensis* (MHOM/BR/76/MA-76), obtained from a human case of diffuse leishmaniasis, were maintained in Schneider’s Insect Medium (Sigma, USA) supplemented with 10% fetal bovine serum (FBS) (Cultilab, Brazil), penicillin (100 U/mL) and streptomycin (100 µg/mL) (Sigma, USA), at 26 °C. The axenic amastigote forms were obtained through the transformation of the promastigote forms after 7–10 days of culture in Schneider’s Insect Medium, supplemented with 5% fetal calf serum, penicillin (100 U/mL) and pH 5.4 at 32 °C [14].

#### Animals

Eight female BALB/c mice aged 4–6 weeks and weighing around 16–18 g, purchased from the Instituto de Ciência e Tecnologia em Biomodelos (ICTB/FIOCRUZ), Rio de Janeiro were used. All the mice were maintained under controlled temperatures and received food and water ad libitum during the experiments.

#### Cell culture

BALB/c mice were inoculated with 3 mL of 3% sodium thioglycolate via intraperitoneal injection. After 72 h, mice were euthanized with 250 µL intraperitoneal injection of a 1:1 mixture of ketamine (100 mg/mL; Syntec, BRA) and xylazine (20 mg/mL; Syntec, BRA). The macrophages were harvested from the peritoneal cavity with PBS solution, centrifuged at 4000 rpm and suspended in RPMI 1640 medium (Sigma, USA), supplemented with 10% FBS, penicillin (100 U/mL) and streptomycin (100 µg/mL), at 37 °C and 5% CO_2_.

#### Activity against axenic amastigote forms of *L. amazonensis*

100 µL of amastigote forms from the axenic culture of *L. amazonensis* were placed into a 96-well plate at 1 × 10^6^ parasites/mL. A total of 100 µL of culture medium with different concentrations (480–3.7 μg/mL) of *M. citrifolia* fruit juice was added to each well and the plate was incubated at 26 °C. Wells with only parasites were kept as control. Amastigote viability was measured at 24, 48 and 72 h by colorimetric assay with MTT [8] to calculate the inhibitory concentration of 50% (IC_50_). Amphotericin B was used as a reference drug.

#### Cytotoxicity and selective index (SI)

BALB/c peritoneal macrophages were cultured in 96-well plate (5 × 10^5^ cells/mL) with different concentrations of *M. citrifolia* fruit juice (2000–3.9 µg/mL), or amphotericin B, to a final volume of 200 µL per well, at 37 °C and 5% CO_2_. Wells without cells were used as blanks and wells with cells only were used as control. After 24 h, parasite viability was evaluated by the modified colorimetric method with tetrazolium-dye 3-(4,5-dimethyl-2-thiazolyl)-2,5-diphenyl-2H-tetrazolium bromide (MTT) (Sigma, USA) to calculate cytotoxicity of 50% (CC_50_) [7]. The selectivity index (SI) was calculated from the ratio of the CC_50_ and the IC_50_ of the axenic amastigote forms.

#### Hydrogen peroxide quantification in culture supernatant from peritoneal macrophages infected with *L. amazonensis* and treated with *M. citrifolia* fruit juice

Peritoneal macrophages from BALB/c were cultured in 96-well plate (5 × 10^6^ cells/well), at 37 °C and 5% CO_2_. A group of cells were infected with *L. amazonensis* promastigote forms, 10:1 parasite/cell, overnight and washed three times with PBS to remove non-internalized parasites. The non-infected cells were treated with different concentrations (125, 250 and 500 µg/mL) of *M. citrifolia* fruit juice for 48 h. The infected cells were treated with 500 µg/mL of *M. citrifolia* fruit juice. Non-infected cells and those treated with phorbol myristate acetate (PMA) (Sigma, USA) 0.2 µM were used as positive controls, and non-treated cells were used as negative controls. After the treatment, the RPMI medium was removed and 100 µL of phenol red medium: 140 mM NaCl (Sigma, USA); 10 mM potassium phosphate buffer pH 7.0; 5.5 mM dextrose (Sigma, USA); 0.56 mM of phenol red (Sigma, USA) and 0.01 mg/mL horseradish peroxidase type II (Sigma, USA) were added. The plates were maintained at 37 °C and 5% CO_2_ and after 1 h the reactions were stopped with 50 µL 5 N NaOH and the absorbance was read at 620 nm [15, 16]. A standard curve with known concentrations of H_2_O_2_ (100–1.5 µM) was used to determine the production of H_2_O_2_ by peritoneal macrophages.

#### Statistical analysis

Data were expressed as mean ± standard deviation from three independent experiments performed at least in quintuplicate analyzed by the Kruskal–Wallis test and Dunn’s multiple comparison test. Statistical analysis were performed with GraphPad Prism 6.01 software.

### Results and discussion

The phytochemical screening results of the lyophilized material revealed the presence of all compounds analyzed (Table 1), confirming the rich chemical composition of the noni juice. That is a novel description and, as a qualitative analysis, the phytochemical screening allowed to clearly demonstrate the diversity of chemical class constituents of *M. citrifolia* fruit juice that has been described in several studies and is responsible for all its pharmacological activities.

**Fig. 1:**
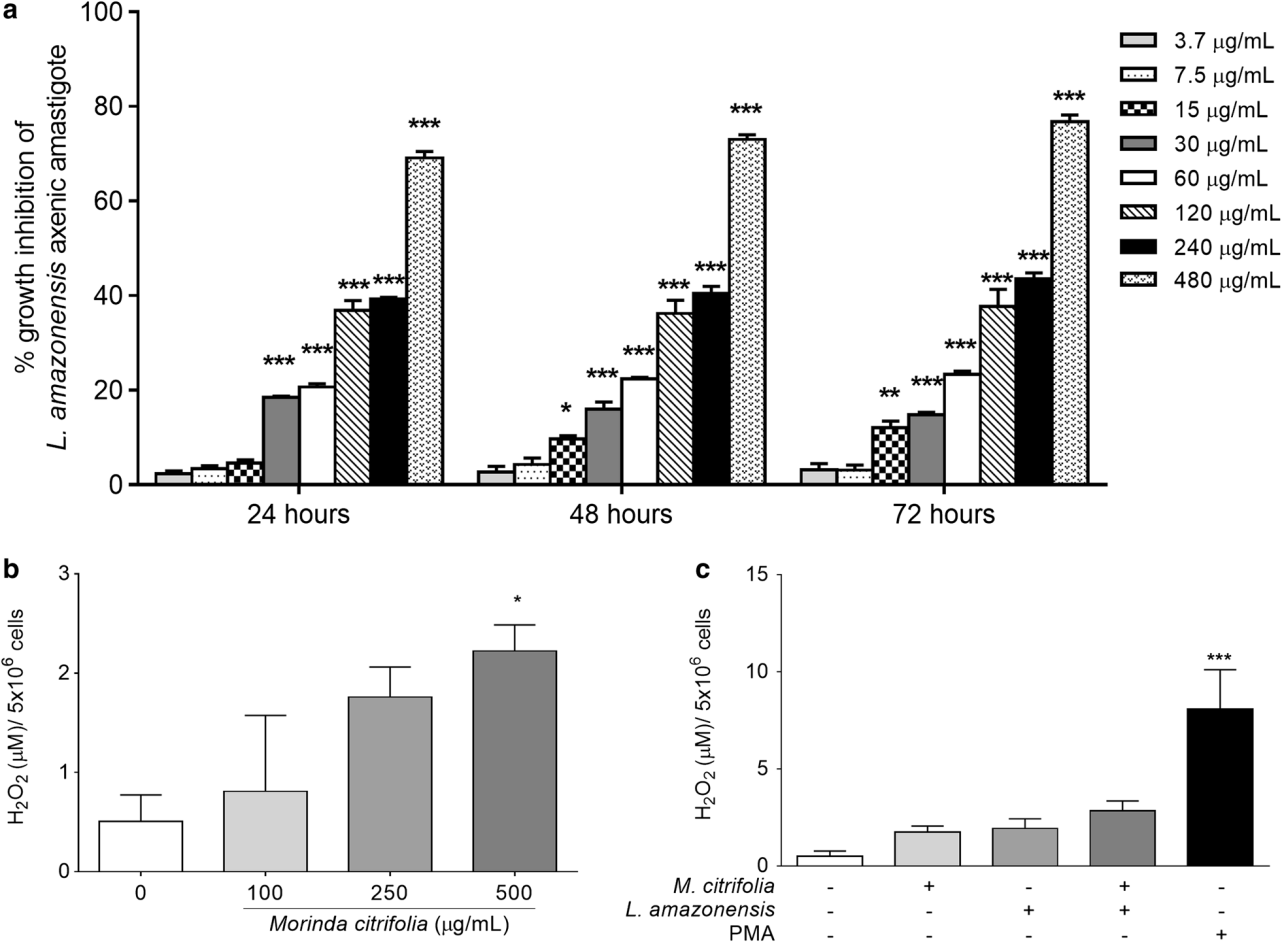
Antileishmanial activity and H_2_O_2_ modulation by *Morinda citrifolia*. **a** Growth inhibition of axenic amastigote forms of *Leishmania amazonensis* and treated with *M. citrifolia* fruit juice. **b** Peritoneal macrophages from BALB/c treated with juice for 48 h increases hydrogen peroxide production. **c** Peritoneal macrophages from BALB/c infected with *L. amazonensis* did not statistically alter H_2_O_2_ production after 48 h of treatment with *M. citrifolia* fruit juice at 500 µg/mL. Data are expressed as mean ± SD and are representative of at least three independent experiments carried out in at least quintuplicate (n = 5); *p < 0.05, **p < 0.01, ***p < 0.001 when compared with untreated group by Kruskal–Wallis test and Dunn’s multiple comparison test

As axenic amastigote is not flagellum-free and motility is not a parameter for assessing its viability, in vitro activity against axenic amastigote was identified by modified colorimetric assay based on MTT reduction [17]. While antileishmanial activity assay with *M. citrifolia* fruit juice revealed dose-dependent activity, time-dependent activity was not observed (Fig. 1a). The IC_50_ of axenic amastigote forms treated with noni juice occurred at a concentration of 240.1 µg/mL. Using the same methodology, the IC_50_ for the axenic amastigote forms was lower than for the previously obtained *L. amazonensis* promastigotes treated with *M. citrifolia* fruit juice (275.3 µg/mL) [8].

Indeed, the differences between the promastigote and amastigote forms are not only morphological, and elucidate the differences in the activity of certain substances to each form. There are differences in the expression of the surface membrane molecules from the promastigote and amastigote forms of *L. tropica* [18]. In *L. mexicana*, there was a difference in the NF-kB modulation of macrophages by both parasite forms. Promastigote forms cleave the p65 subunit, generating a small p35 subunit, while the amastigote forms completely degrade the p65 subunit, without the production of a p35 subunit, and subsequently no IFN-γ and nitric oxide production occurs [19]. There are also metabolic differences between the two forms. The in vitro culture of amastigote forms has a higher consumption of fatty acids from the culture medium than promastigote forms in the logarithmic growth phase [20, 21].

The results of the present study corroborate previous studies which identified *M. citrifolia* activity against promastigote and intracellular amastigote forms of *L. amazonensis*. *M. citrifolia* activity against intracellular amastigotes showed a combined effect, direct and indirect, of juice on *L. amazonensis*. To have access to the intracellular parasite, the antileishmanial compounds must be able to cross the cell membranes to act directly on the parasite inside the vacuole. The effectiveness against axenic forms, out of cells, demonstrates the direct effect of *M. citrifolia* juice, since all the constituents are bioavailable to direct act on the parasite.

The cytotoxicity assay showed that noni did not present toxicity for cells at the concentrations tested, while amphotericin B exhibited a CC_50_ of 3.1 µg/mL. The cytotoxicity still allowed the SI to be obtained for axenic amastigote forms. The SI of *M. citrifolia* fruit juice demonstrated 8.3 times more toxicity for parasites than for the *L. amazonensis* axenic amastigote form, while the reference drug amphotericin B presented an SI of 1.0. These results are similar to the findings of previous research using intracellular amastigotes and amphotericin B, where *M. citrifolia* exhibited a higher SI than amphotericin B [8], demonstrating the safe use of *M. citrifolia*.

The analysis of IC_50_ demonstrated the difference between the axenic cultures of *L. amazonensis* forms and the intracellular form. The IC_50_ for axenic amastigotes, as well as the activity against the promastigote cultured in the axenic form, also differs from that previously found for the intracellular amastigote (208.4 µg/mL). The increase in the activity of *M. citrifolia* fruit juice against the intracellular amastigote is related to macrophage modulation and the induction of NO production, a potent microbicide against intracellular parasites such as *Leishmania* [8]. Nevertheless, other mechanisms of macrophage activation may be involved in *M. citrifolia* antileishmanial activity.

In its life cycle, the *Leishmania* parasite differentiates from the promastigote to amastigote forms within macrophages in a parasitophorous vacuole where it is exposed to continuous ROS, such as H_2_O_2_, and NO [11], generating acute oxidative stress. Oxidative stress is very harmful to telomeres and induces DNA damage in *Leishmania* species such as *L. amazonensis* [22]. It was demonstrated that while production of ROS is involved in the killing of *L. braziliensis* in human cutaneous leishmaniasis, NO alone is not sufficient to control infection and may contribute to tissue damage [23]. Therefore, to identify other forms of macrophage activation by *M. citrifolia* fruit juice, the H_2_O_2_ was quantified in supernatant of BALB/c peritoneal macrophages treated with this juice.

Noni juice has already showed absence of endotoxin that ensures no endotoxin interference in the data obtained [8]. It was then observed that the juice induced H_2_O_2_ production in BALB/c peritoneal macrophages in a dose-dependent manner. H_2_O_2_ quantification showed that at 500 µg/mL *M. citrifolia* fruit juice induces the production of H_2_O_2_ in BALB/c peritoneal macrophages at 4.4 times the rate of untreated cells (Fig. 1b). Hydrogen peroxide, like other ROS and reactive species of nitrogen, is a chemically reactive micromolecule that does not discriminate the genomic source of its chemical targets, as part of the nonspecific immune system, and participates in the response against intracellular microorganisms [10].

Although H_2_O_2_ induction may be important for various infections, and is responsible for the antileishmanial activity of nerve growth factor in a murine model infected with *Leishmania donovani* [24], treatment with *M. citrifolia* fruit juice did not induce H_2_O_2_ production in macrophages infected with *L. amazonensis*. The production of H_2_O_2_ was statistically equal between infected macrophages treated or untreated with fruit juice (Fig. 1c).

**Fig. 2:**
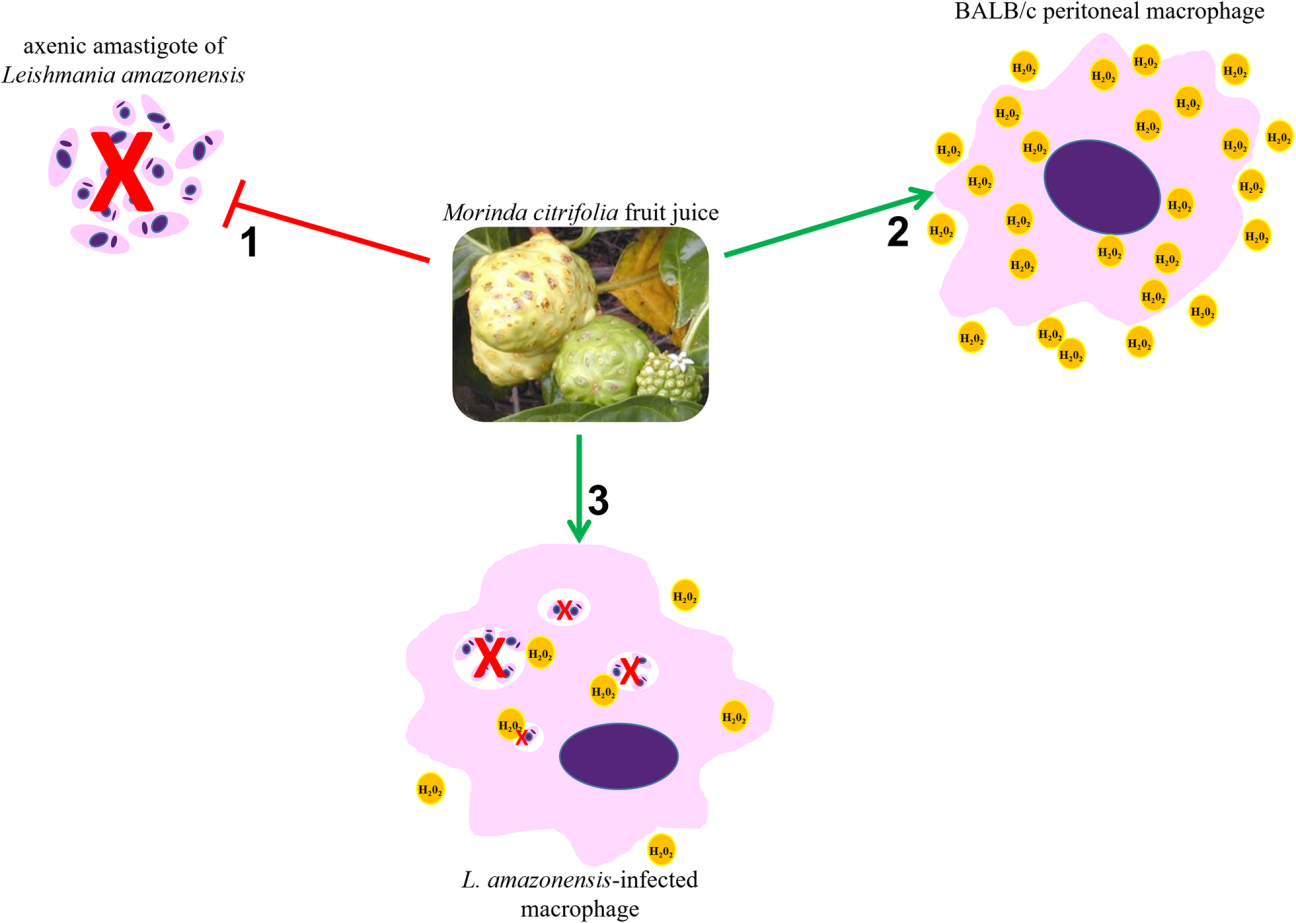
Activity against *Leishmania amazonensis* axenic amastigote forms and hydrogen peroxide induction by *Morinda citrifolia* fruit juice. *M. citrifolia* treatment induces death of axenic amastigotes forms of *L. amazonensis* (1); and increases hydrogen peroxide and NO production by peritoneal macrophages (2) but not hydrogen peroxide in *Leishmania*-infected peritoneal macrophages (3)

It is known that intracellular macrophage pathogen-like *Leishmania* inhibits oxidative burst-mediated macrophage apoptosis to protect its survival and replication niche, as one of the mechanisms for establishing infection. *L. donovani*, for example, prevents the oxidative burst-mediated apoptosis of host macrophages through the selective induction of suppressors of cytokine signaling proteins [25]. Moreover, acute oxidative stress by ROS caused the death of some of the *L. amazonensis* population, although the remaining parasites induced cell cycle arrest in the G2/M phase, which could continue to proliferate and replicate the DNA and became more resistant to oxidative stress [21]. Thereby, although H_2_O_2_ does not play an important role in its antileishmanial activity, *M. citrifolia* fruit juice can induce H_2_O_2_ production in BALB/c peritoneal macrophages (Fig. 2). Further studies are necessary to understand how this induction may be related to other activities of *M. citrifolia*.

## Limitations

The main limitation and highlight of this study is the use of axenic amastigote forms to assess antileishmanial. The activity against axenic amastigote forms demonstrates the direct action of juice on the parasite, corroborate and assert the antileishmanial activity of *M. citriflia* fruit juice, giving robustness to the results described previously, and justifying further studies to clarify the potential of *M. citrifolia* as an alternative treatment for animals and humans or even use in association against leishmaniasis. Finally, the hydrogen peroxide induction by *M. citriflia* fruit juice in BALB/c peritoneal macrophages, as well other ROS not evaluated in this study, indicates a possible route to macrophage activation and immunomodulatory effects that can be investigated in further studies.

## Abbreviations

IC50: inhibitory concentration to 50%
NO: nitric oxide
iNOS: nitric oxide synthase inducible
H_2_O_2_: hydrogen peroxide
ROS: reactive oxygen species
FBS: fetal bovine serum
CC50: cytotoxicity concentration to 50%
MTT: tetrazolium-dye 3-(4,5-dimethyl-2-thiazolyl)-2,5-diphenyl-2H-tetrazolium bromide
DMSO: dimethyl sulfoxide
PMA: phorbol myristate acetate
